# 1-Meth­oxy-4-methyl-9,10-anthraquinone

**DOI:** 10.1107/S1600536811041912

**Published:** 2011-10-22

**Authors:** Che Puteh Osman, Rohaya Ahmad, Nor Hadiani Ismail, Khalijah Awang, Seik Weng Ng

**Affiliations:** a Faculty of Applied Sciences, Universiti Teknologi MARA, 40450 Shah Alam, Malaysia; b Department of Chemistry, University of Malaya, 50603 Kuala Lumpur, Malaysia; c Chemistry Department, Faculty of Science, King Abdulaziz University, PO Box 80203 Jeddah, Saudi Arabia

## Abstract

The non-H atoms of the title compound, C_16_H_12_O_3_, lie approximately in a common plane (r.m.s. deviation = 0.032 Å). The methyl C atom is forced away from the carbonyl O atom which can be seen by the widened C_fused ring_–C_benzene_–C_meth­yl_ angle of 125.8 (2)°.

## Related literature

For the synthesis, see: Bentley *et al.* (1907 ▶); Fischer & Ziegler (1913 ▶).
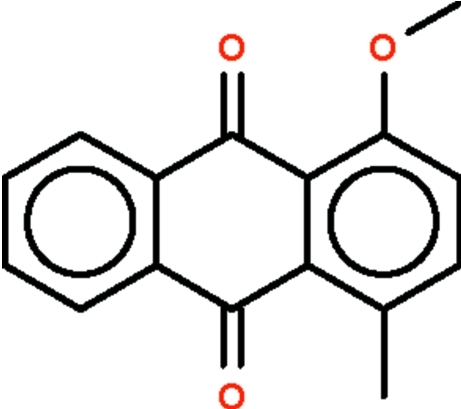


## Experimental

### 

#### Crystal data

C_16_H_12_O_3_
*M* *_r_* = 252.26
Monoclinic, 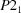

*a* = 8.8808 (4) Å
*b* = 4.8940 (2) Å
*c* = 13.7792 (5) Å
β = 96.136 (4)°
*V* = 595.45 (4) Å^3^
*Z* = 2
Cu *K*α radiation
μ = 0.79 mm^−1^
*T* = 100 K
0.30 × 0.10 × 0.02 mm

#### Data collection

Agilent SuperNova Dual diffractometer with an Atlas detector
Absorption correction: multi-scan (*CrysAlis PRO*; Agilent, 2010 ▶) *T*_min_ = 0.797, *T*_max_ = 0.984
2343 measured reflections
1367 independent reflections
1317 reflections with *I* > 2σ(*I*)
*R*_int_ = 0.014

#### Refinement

*R*[*F* ^2^ > 2σ(*F* ^2^)] = 0.036
*wR*(*F* ^2^) = 0.108
*S* = 1.16
1367 reflections
173 parameters
1 restraint
H-atom parameters constrained
Δρ_max_ = 0.19 e Å^−3^
Δρ_min_ = −0.18 e Å^−3^

### 

Data collection: *CrysAlis PRO* (Agilent, 2010 ▶); cell refinement: *CrysAlis PRO*; data reduction: *CrysAlis PRO*; program(s) used to solve structure: *SHELXS97* (Sheldrick, 2008 ▶); program(s) used to refine structure: *SHELXL97* (Sheldrick, 2008 ▶); molecular graphics: *X-SEED* (Barbour, 2001 ▶); software used to prepare material for publication: *publCIF* (Westrip, 2010 ▶).

## Supplementary Material

Crystal structure: contains datablock(s) global, I. DOI: 10.1107/S1600536811041912/bt5675sup1.cif

Structure factors: contains datablock(s) I. DOI: 10.1107/S1600536811041912/bt5675Isup2.hkl

Supplementary material file. DOI: 10.1107/S1600536811041912/bt5675Isup3.cml

Additional supplementary materials:  crystallographic information; 3D view; checkCIF report

## Figures and Tables

**Fig. 1.**
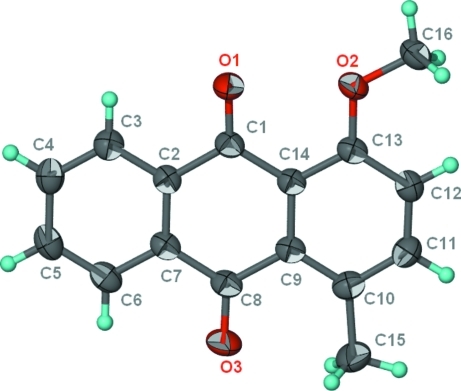
Anisotropic displacement ellipsoid plot (Barbour, 2001) of C16H12O3 at the 70% probability level; hydrogen atoms are drawn as spheres of arbitrary radius.

## supplementary crystallographic information

### Comment

1-Methoxy-4-methyl-9,10-anthraquinone was reported more than a century ago (Bentley *et al.*, 1907; Fischer & Ziegler, 1913). We have used a modification of the synthesis to prepare 1-hydroxy-4-methyl-9,10-anthraquinone, which was then methylated to yield the title compound (Scheme). The non-hydrogen atoms of 1-methyl-4-methoxy-9,10-anthraquinone lie on a plane (r.m.s. deviation 0.032Å). The methyl C atom is forced away from the by the carbonyl O atom that is four bonds removed (C_fused ring_–C_benzene_–C_methyl_ 125.8 (2) °].

Its isolation from plants has not been reported yet.

### Experimental

Phthalic anhydride (1.00 g, 0.67 mmol) and *p*-cresol (1.63 g, 1.50 mmol) were heated in a mixture of aluminium chloride (45 g) and sodium chloride (9 g) heated to 423–443 K for an hour. The reaction mixture turned deep red. Water (500 ml) containing concentrated hydrochloric acid (15 ml) was added. The product was collected and washed with saturated sodium bicarbonate, and was next purified by medium-pressure liquid chromatography (hexane: ethyl acetate) to give 1-hydroxy-4-methyl-9,10-anthroquinone (60% yield).

In the subsequent methylation reaction, 1-hydroxy-4-methyl-9,10-anthraquinone (1 mmol) and methyl iodide (1.5 mmol) along with potassium carbonate (1 mmol) were heated in acetone (30 ml) for 24 h. The solvent was removed and the product dissolved in dichloromethane. The solution was extraced with water. The organic layer was purified by medium-pressure liquid chromatography (hexane: ethyl acetate) to give the pure title compound (80% yield). Crystals were obtained by using methanol as solvent for recrystallization. The formulation was established by proton and carbon-13 NMR spectroscopy.

### Refinement

H-atoms were placed in calculated positions [C—H 0.95 to 0.98 Å, *U*_iso_(H) 1.2 to 1.5*U*_eq_(C)] and were included in the refinement in the riding model approximation.

As the Friedel pair coverage was only 37%, 412 Friedel pairs were merged.

### Crystal data

**Table:**

C_16_H_12_O_3_	*F*(000) = 264
*M**_r_* = 252.26	*D*_x_ = 1.407 Mg m^−^^3^
Monoclinic, *P*2_1_	Cu *K*α radiation, λ = 1.54184 Å
Hall symbol: P 2yb	Cell parameters from 1483 reflections
*a* = 8.8808 (4) Å	θ = 3.2–76.1°
*b* = 4.8940 (2) Å	µ = 0.79 mm^−^^1^
*c* = 13.7792 (5) Å	*T* = 100 K
β = 96.136 (4)°	Plate, orange
*V* = 595.45 (4) Å^3^	0.30 × 0.10 × 0.02 mm
*Z* = 2	

### Data collection

**Table:**

Agilent SuperNova Dual diffractometer with an Atlas detector	1367 independent reflections
Radiation source: SuperNova (Cu) X-ray Source	1317 reflections with *I* > 2σ(*I*)
Mirror	*R*_int_ = 0.014
Detector resolution: 10.4041 pixels mm^-1^	θ_max_ = 76.3°, θ_min_ = 3.2°
ω scans	*h* = −7→11
Absorption correction: multi-scan (*CrysAlis PRO*; Agilent, 2010)	*k* = −5→6
*T*_min_ = 0.797, *T*_max_ = 0.984	*l* = −17→17
2343 measured reflections	

### Refinement

**Table:**

Refinement on *F*^2^	Primary atom site location: structure-invariant direct methods
Least-squares matrix: full	Secondary atom site location: difference Fourier map
*R*[*F*^2^ > 2σ(*F*^2^)] = 0.036	Hydrogen site location: inferred from neighbouring sites
*wR*(*F*^2^) = 0.108	H-atom parameters constrained
*S* = 1.16	*w* = 1/[σ^2^(*F*_o_^2^) + (0.0706*P*)^2^ + 0.0361*P*] where *P* = (*F*_o_^2^ + 2*F*_c_^2^)/3
1367 reflections	(Δ/σ)_max_ = 0.001
173 parameters	Δρ_max_ = 0.19 e Å^−^^3^
1 restraint	Δρ_min_ = −0.18 e Å^−^^3^

### Fractional atomic coordinates and isotropic or equivalent isotropic displacement parameters (Å^2^)

**Table:**

	*x*	*y*	*z*	*U*_iso_*/*U*_eq_	
O1	0.55994 (14)	0.5010 (4)	0.81572 (9)	0.0371 (4)	
O2	0.66956 (14)	0.8795 (3)	0.93147 (9)	0.0317 (3)	
O3	1.07132 (15)	0.4573 (4)	0.64116 (10)	0.0390 (4)	
C1	0.67730 (18)	0.5016 (4)	0.77647 (11)	0.0248 (4)	
C2	0.69140 (19)	0.3128 (4)	0.69352 (11)	0.0240 (4)	
C3	0.5701 (2)	0.1401 (5)	0.66264 (12)	0.0291 (4)	
H3	0.4802	0.1455	0.6943	0.035*	
C4	0.5813 (2)	−0.0384 (5)	0.58604 (13)	0.0323 (4)	
H4	0.4992	−0.1568	0.5655	0.039*	
C5	0.7123 (2)	−0.0457 (5)	0.53870 (13)	0.0324 (4)	
H5	0.7185	−0.1666	0.4853	0.039*	
C6	0.8330 (2)	0.1223 (5)	0.56916 (11)	0.0300 (4)	
H6	0.9226	0.1160	0.5372	0.036*	
C7	0.82303 (19)	0.3018 (4)	0.64715 (11)	0.0247 (4)	
C8	0.95447 (19)	0.4775 (4)	0.68049 (12)	0.0264 (4)	
C9	0.94200 (19)	0.6767 (4)	0.76192 (11)	0.0235 (4)	
C10	1.06468 (19)	0.8520 (4)	0.79051 (12)	0.0285 (4)	
C11	1.0481 (2)	1.0335 (4)	0.86625 (13)	0.0304 (4)	
H11	1.1295	1.1535	0.8865	0.037*	
C12	0.9187 (2)	1.0470 (4)	0.91328 (13)	0.0280 (4)	
H12	0.9124	1.1755	0.9643	0.034*	
C13	0.79734 (18)	0.8729 (4)	0.88619 (11)	0.0250 (4)	
C14	0.80695 (18)	0.6854 (4)	0.80882 (11)	0.0233 (4)	
C15	1.2125 (2)	0.8622 (6)	0.74564 (15)	0.0400 (5)	
H15A	1.1927	0.9088	0.6763	0.060*	
H15B	1.2622	0.6833	0.7524	0.060*	
H15C	1.2786	1.0010	0.7791	0.060*	
C16	0.6594 (2)	1.0809 (5)	1.00589 (13)	0.0334 (4)	
H16A	0.5623	1.0618	1.0330	0.050*	
H16B	0.6663	1.2637	0.9777	0.050*	
H16C	0.7425	1.0549	1.0578	0.050*	

### Atomic displacement parameters (Å^2^)

**Table:**

	*U*^11^	*U*^22^	*U*^33^	*U*^12^	*U*^13^	*U*^23^
O1	0.0319 (6)	0.0462 (9)	0.0358 (7)	−0.0103 (7)	0.0162 (5)	−0.0114 (7)
O2	0.0325 (6)	0.0349 (8)	0.0297 (6)	−0.0025 (6)	0.0130 (5)	−0.0066 (6)
O3	0.0362 (7)	0.0443 (9)	0.0403 (7)	−0.0043 (7)	0.0218 (6)	−0.0053 (7)
C1	0.0282 (7)	0.0262 (9)	0.0213 (7)	−0.0011 (8)	0.0086 (6)	0.0027 (8)
C2	0.0290 (8)	0.0234 (9)	0.0198 (7)	0.0005 (8)	0.0039 (6)	0.0035 (7)
C3	0.0328 (8)	0.0297 (10)	0.0249 (7)	−0.0020 (9)	0.0040 (6)	0.0031 (8)
C4	0.0390 (9)	0.0294 (10)	0.0274 (8)	−0.0019 (9)	−0.0019 (7)	−0.0005 (9)
C5	0.0439 (10)	0.0288 (10)	0.0241 (8)	0.0043 (10)	0.0019 (7)	−0.0019 (9)
C6	0.0377 (9)	0.0304 (10)	0.0226 (7)	0.0059 (9)	0.0070 (6)	0.0019 (8)
C7	0.0311 (8)	0.0240 (9)	0.0197 (7)	0.0029 (8)	0.0065 (6)	0.0051 (7)
C8	0.0310 (8)	0.0261 (9)	0.0239 (7)	0.0013 (9)	0.0107 (6)	0.0050 (9)
C9	0.0271 (7)	0.0222 (9)	0.0220 (7)	0.0009 (8)	0.0064 (6)	0.0064 (8)
C10	0.0273 (8)	0.0301 (10)	0.0286 (8)	−0.0005 (8)	0.0055 (6)	0.0064 (8)
C11	0.0300 (8)	0.0294 (11)	0.0316 (9)	−0.0050 (8)	0.0018 (7)	0.0021 (8)
C12	0.0333 (8)	0.0260 (10)	0.0245 (7)	0.0011 (8)	0.0018 (6)	0.0003 (8)
C13	0.0276 (8)	0.0258 (9)	0.0222 (7)	0.0029 (8)	0.0060 (6)	0.0042 (8)
C14	0.0262 (7)	0.0232 (9)	0.0212 (7)	0.0007 (7)	0.0056 (6)	0.0036 (7)
C15	0.0322 (9)	0.0474 (13)	0.0420 (10)	−0.0088 (10)	0.0107 (8)	−0.0021 (11)
C16	0.0387 (9)	0.0321 (11)	0.0309 (8)	0.0016 (9)	0.0105 (7)	−0.0045 (8)

### Geometric parameters (Å, °)

**Table:**

O1—C1	1.224 (2)	C8—C9	1.499 (3)
O2—C13	1.3526 (18)	C9—C10	1.409 (3)
O2—C16	1.432 (2)	C9—C14	1.422 (2)
O3—C8	1.225 (2)	C10—C11	1.390 (3)
C1—C2	1.485 (2)	C10—C15	1.511 (2)
C1—C14	1.491 (3)	C11—C12	1.380 (2)
C2—C7	1.392 (2)	C11—H11	0.9500
C2—C3	1.399 (3)	C12—C13	1.393 (3)
C3—C4	1.382 (3)	C12—H12	0.9500
C3—H3	0.9500	C13—C14	1.416 (2)
C4—C5	1.394 (3)	C15—H15A	0.9800
C4—H4	0.9500	C15—H15B	0.9800
C5—C6	1.380 (3)	C15—H15C	0.9800
C5—H5	0.9500	C16—H16A	0.9800
C6—C7	1.398 (3)	C16—H16B	0.9800
C6—H6	0.9500	C16—H16C	0.9800
C7—C8	1.483 (3)		
			
C13—O2—C16	117.84 (15)	C11—C10—C9	117.28 (16)
O1—C1—C2	118.97 (17)	C11—C10—C15	116.89 (18)
O1—C1—C14	122.39 (16)	C9—C10—C15	125.83 (18)
C2—C1—C14	118.64 (14)	C12—C11—C10	122.90 (18)
C7—C2—C3	119.60 (17)	C12—C11—H11	118.6
C7—C2—C1	121.43 (15)	C10—C11—H11	118.6
C3—C2—C1	118.96 (15)	C11—C12—C13	120.28 (18)
C4—C3—C2	119.89 (17)	C11—C12—H12	119.9
C4—C3—H3	120.1	C13—C12—H12	119.9
C2—C3—H3	120.1	O2—C13—C12	121.67 (16)
C3—C4—C5	120.35 (18)	O2—C13—C14	118.93 (15)
C3—C4—H4	119.8	C12—C13—C14	119.39 (15)
C5—C4—H4	119.8	C13—C14—C9	118.89 (15)
C6—C5—C4	120.18 (18)	C13—C14—C1	120.61 (14)
C6—C5—H5	119.9	C9—C14—C1	120.49 (15)
C4—C5—H5	119.9	C10—C15—H15A	109.5
C5—C6—C7	119.78 (17)	C10—C15—H15B	109.5
C5—C6—H6	120.1	H15A—C15—H15B	109.5
C7—C6—H6	120.1	C10—C15—H15C	109.5
C2—C7—C6	120.19 (17)	H15A—C15—H15C	109.5
C2—C7—C8	120.47 (16)	H15B—C15—H15C	109.5
C6—C7—C8	119.34 (15)	O2—C16—H16A	109.5
O3—C8—C7	119.47 (18)	O2—C16—H16B	109.5
O3—C8—C9	121.16 (18)	H16A—C16—H16B	109.5
C7—C8—C9	119.36 (14)	O2—C16—H16C	109.5
C10—C9—C14	121.24 (16)	H16A—C16—H16C	109.5
C10—C9—C8	119.22 (15)	H16B—C16—H16C	109.5
C14—C9—C8	119.54 (15)		
			
O1—C1—C2—C7	−179.01 (17)	C14—C9—C10—C11	0.0 (3)
C14—C1—C2—C7	1.1 (2)	C8—C9—C10—C11	179.94 (16)
O1—C1—C2—C3	0.0 (3)	C14—C9—C10—C15	−179.51 (18)
C14—C1—C2—C3	−179.87 (16)	C8—C9—C10—C15	0.5 (3)
C7—C2—C3—C4	−0.5 (3)	C9—C10—C11—C12	0.1 (3)
C1—C2—C3—C4	−179.55 (17)	C15—C10—C11—C12	179.60 (18)
C2—C3—C4—C5	−0.6 (3)	C10—C11—C12—C13	0.5 (3)
C3—C4—C5—C6	1.1 (3)	C16—O2—C13—C12	2.8 (3)
C4—C5—C6—C7	−0.6 (3)	C16—O2—C13—C14	−176.68 (15)
C3—C2—C7—C6	1.0 (3)	C11—C12—C13—O2	179.38 (16)
C1—C2—C7—C6	−179.92 (16)	C11—C12—C13—C14	−1.1 (3)
C3—C2—C7—C8	−178.30 (16)	O2—C13—C14—C9	−179.34 (15)
C1—C2—C7—C8	0.7 (3)	C12—C13—C14—C9	1.1 (2)
C5—C6—C7—C2	−0.5 (3)	O2—C13—C14—C1	1.1 (2)
C5—C6—C7—C8	178.84 (17)	C12—C13—C14—C1	−178.41 (16)
C2—C7—C8—O3	177.22 (17)	C10—C9—C14—C13	−0.6 (2)
C6—C7—C8—O3	−2.1 (3)	C8—C9—C14—C13	179.45 (16)
C2—C7—C8—C9	−2.7 (3)	C10—C9—C14—C1	178.98 (16)
C6—C7—C8—C9	177.98 (16)	C8—C9—C14—C1	−1.0 (2)
O3—C8—C9—C10	2.9 (3)	O1—C1—C14—C13	−1.3 (3)
C7—C8—C9—C10	−177.18 (16)	C2—C1—C14—C13	178.62 (15)
O3—C8—C9—C14	−177.11 (16)	O1—C1—C14—C9	179.17 (18)
C7—C8—C9—C14	2.8 (2)	C2—C1—C14—C9	−0.9 (2)

## References

[bb1] Agilent (2010). *CrysAlis PRO* Agilent Technologies, Yarnton, England.

[bb2] Barbour, L. J. (2001). *J. Supramol. Chem.* **1**, 189–191.

[bb3] Bentley, W. H., Gardner, H. D. & Weizmann, C. (1907). *J. Chem. Soc. Trans.* **91**, 1626–1640.

[bb4] Fischer, O. & Ziegler, H. (1913). *J. Prakt. Chem.* **86**, 289–297.

[bb5] Sheldrick, G. M. (2008). *Acta Cryst.* A**64**, 112–122. 10.1107/S0108767307043930 18156677

[bb6] Westrip, S. P. (2010). *J. Appl. Cryst.* **43**, 920–925.

